# The Relationship Between the Vaginal Microbiota and the Ovarian Cancer Microenvironment: A Journey from Ideas to Insights

**DOI:** 10.3390/cells14201590

**Published:** 2025-10-13

**Authors:** Stefano Restaino, Giulia Pellecchia, Martina Arcieri, Eva Pericolini, Giorgio Bogani, Alice Poli, Federico Paparcura, Sara Pregnolato, Doriana Armenise, Barbara Frossi, Gianluca Tell, Carlo Tascini, Lorenza Driul, Anna Biasioli, Vito Andrea Capozzi, Carlo Ronsini, Luigi Della Corte, Canio Martinelli, Alfredo Ercoli, Francesco De Seta, Giuseppe Vizzielli

**Affiliations:** 1 Department of Maternal and Child Health, Obstetrics and Gynecology Clinic, University Hospital of Udine, 33100 Udine, Italy; stefano.restaino@asufc.sanita.fvg.it (S.R.); pellecchia.giulia001@spes.uniud.it (G.P.); poli.alice@spes.uniud.it (A.P.); paparcura.federico@spes.uniud.it (F.P.); pregnolato.sara@spes.uniud.it (S.P.); armenise.doriana@spes.uniud.it (D.A.); lorenza.driul@uniud.it (L.D.); anna.biasioli@asufc.sanita.fvg.it (A.B.); giuseppe.vizzielli@uniud.it (G.V.); 2 PhD School in Biomedical Sciences, Gender Medicine, Child and Women Health, University of Sassari, 07100 Sassari, Italy; 3 Department of Medicine (DMED), University of Udine, 33100 Udine, Italy; barbara.frossi@uniud.it; 4 Department of Surgical, Medical, Dental and Morphological Sciences with Interest in Transplant, Oncological and Regenerative Medicine, University of Modena and Reggio Emilia, 41125 Modena, Italy; eva.pericolini@unimore.it; 5 Gynaecological Oncology Unit, Fondazione IRCCS Istituto Nazionale dei Tumori di Milano, 20133 Milano, Italy; giorgio.bogani@istitutotumori.mi.it; 6 Molecular Biology Section, Department of Medicine, University of Udine, 33100 Udine, Italy; gianluca.tell@spes.uniud.it; 7 Infectious Diseases Clinic, Azienda Sanitaria Universitaria Friuli Centrale, 33100 Udine, Italy; carlo.tascini@uniud.it; 8 Department of Obstetrics and Gynecology, University of Parma, 43125 Parma, Italy; capozzivitoandrea@gmail.com; 9 Unit of Gynecologic Oncology, National Cancer Institute, IRCCS, Fondazione “G. Pascale”, 80131 Naples, Italy; carlo.ronsini@unicampania.it; 10 Department of Neuroscience, Reproductive Sciences and Dentistry, School of Medicine, University of Naples “Federico II”, 80131 Naples, Italy; dellacorte.luigi25@gmail.com; 11 Unit of Gynecology and Obstetrics, Department of Human Pathology of Adults and Developmental Age, Gaetano Martino University Hospital, 98124 Messina, Italy; canio.martinelli@temple.edu (C.M.); alfredo.ercoli@unime.it (A.E.); 12 Sbarro Institute for Cancer Research and Molecular Medicine and Center for Biotechnology, College of Science and Technology, Temple University, Philadelphia, PA 19122, USA; 13 Department of Medical Sciences, University of Trieste, 34127 Trieste, Italy; fradeseta@gmail.com

**Keywords:** microenvironment, tumor, genital neoplasms, female/prevention & control, microbiota

## Abstract

**Background:** The tumor microenvironment offers a new perspective in gynecologic oncology. In ovarian cancer, numerous preclinical studies, especially organoid models, have highlighted cellular, immune, and biochemical mechanisms. Beyond these sophisticated findings, more practical aspects require attention, such as the role of vaginal microbiota, which represents an interplay between external agents and internal genitalia, and its potential profiling role in early detection beyond the promise of microbiota-targeted therapies. **Objectives**: This review aims to assess whether such a correlation is speculative or scientifically grounded. **Methods**: A focused literature search was conducted on vaginal microbiota and its correlation with ovarian cancer to define the current state of knowledge. **Results**: Mixed outcomes have been reported, yet there is a rational and scientific basis supporting further investigation. Clinical approaches increasingly consider vaginal microbiota as relevant. However, we have to say that most available evidence is still preliminary and largely preclinical to set realistic expectations for readers. Although additional studies are needed, emerging insights highlight its importance and practical implications. We present a diagnostic–therapeutic management flowchart summarizing current evidence). **Discussion**: Most links between the vaginal microbiota and ovarian cancer are correlational rather than causal. The idea that microbes ascend from the vagina to the ovaries is proposed but still definitely not demonstrated. Confounding factors like age, hormones, and BRCA status complicate interpretation, and ovarian cancer itself could secondarily alter the microbiota. Mechanistic studies and longitudinal data are still needed to clarify whether dysbiosis contributes to carcinogenesis or is merely a consequence. As gynecologists, we summarize key aspects and emphasize to colleagues the importance of incorporating these findings into daily clinical practice. Vaginal dysbiosis should be considered not only a local imbalance but also a potential strategy for primary cancer prevention. **Conclusions**: Future research on the tumor microenvironment and vaginal microbiota will expand scientific knowledge and guide innovative preventive and therapeutic strategies.

## 1. Introduction

Ovarian cancer is one of the most common gynecological malignancies, with an estimated incidence of approximately 295,000 new cases worldwide each year [1]. It is often diagnosed at an advanced stage due to the absence of early symptoms, contributing to its high mortality rate. Unfortunately, to date, there are no available screening or prevention methods. Therefore, research must strive to overcome this challenge by enhancing therapeutic strategies.

Immunotherapy and the study of the tumor microenvironment represent new frontiers in this arena, promising increasingly valuable data [2]. Building on these premises, our narrative review aims to understand the current state of the art of the tumor microenvironment in ovarian cancer, focusing on the evidence regarding the influence of the vaginal microbiome (VMB) on it. The VMB impacts both reproductive health and gynecologic cancers. A balanced, Lactobacillus-dominant microbiota supports fertility, reduces the risk of infections, and contributes to healthy pregnancy outcomes. In contrast, microbial dysbiosis is characterized by an overgrowth of pathogenic bacteria such as *Gardnerella vaginalis* and *Atopobium vaginae*, which are associated with infertility, recurrent pregnancy loss, and preterm birth.

Beyond reproduction, the VMB is increasingly recognized for its role in gynecologic cancers. Persistent dysbiosis and chronic inflammation are linked to the progression of cervical, endometrial, and ovarian cancers, likely through immune modulation and synergy with oncogenic viruses like HPV. The presence of specific bacterial signatures may serve as diagnostic biomarkers, opening new avenues for early detection and targeted therapies. The potential for microbiota-based interventions to improve both reproductive and oncologic outcomes is becoming better defined and understood [3].

**Figure 1:**
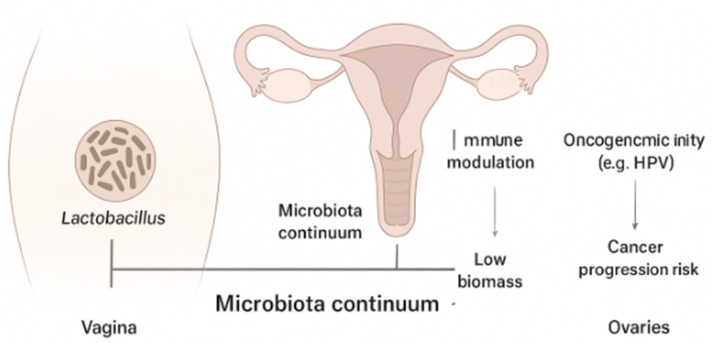
The “microbiome continuum” concept.

In this context, the concept of the “microbiota continuum” from the lower to the upper female reproductive tract (FRT) has been proposed and endorsed, showing decreasing biomass and *Lactobacillus* spp. abundance, along with increasing diversity toward the upper FRT. This suggests the influence the VMB could have on the upper tract [4] (Figure 1).

However, additional studies are needed to clarify the relationship between VMBs and ovarian cancer, which could possibly result in new preventive and therapeutic strategies.

**Figure 2:**
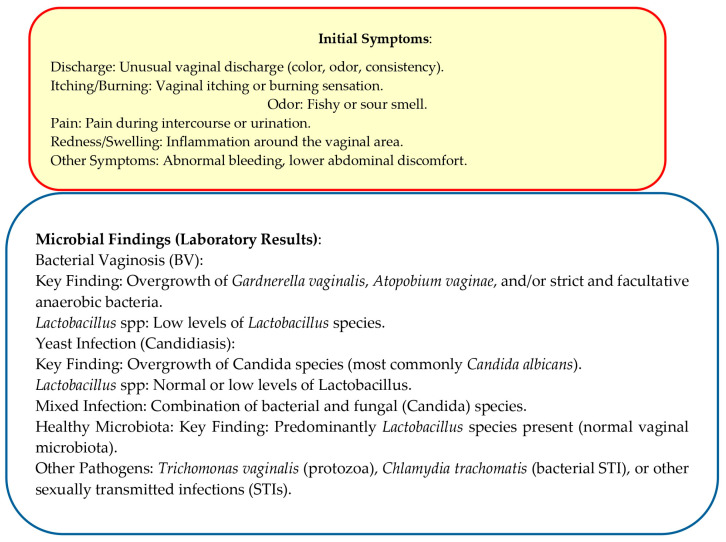

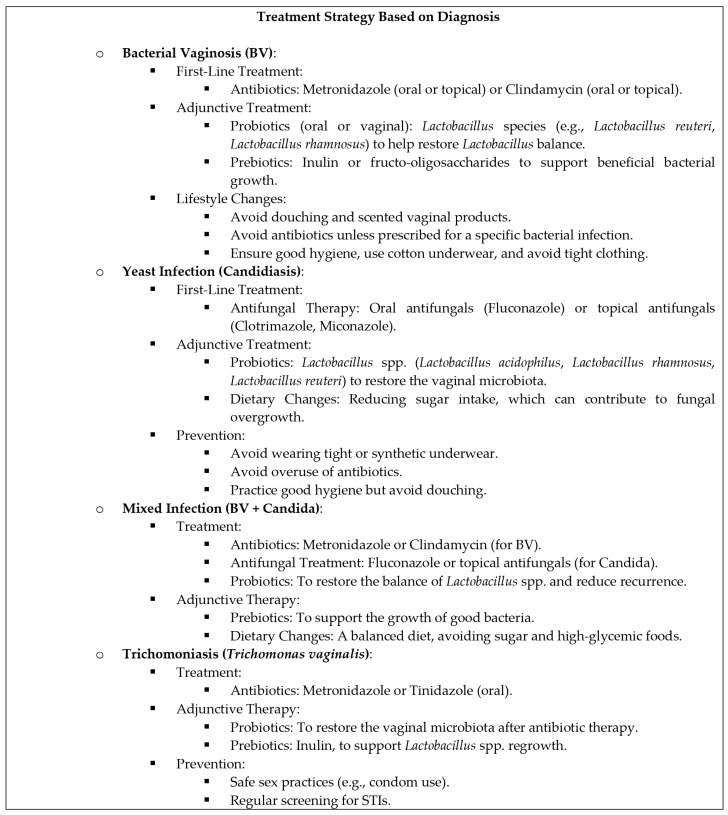

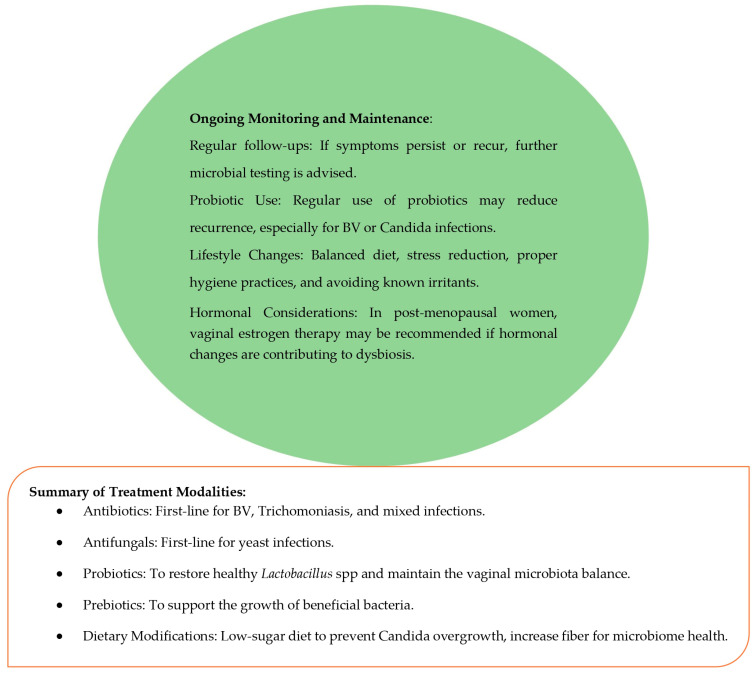
Flowchart for Vaginal Dysbiosis Diagnosis and Treatment.

Therefore, we aim to assess the current state of the art on this issue through the primary scientific evidence from recent years, thus offering clinicians advice and alerts to be considered in their routine clinical practice (Figure 2).

## 2. Materials and Methods

The review was conducted by searching PubMed for results using the search strategies summarized in Table 1 below. Articles were screened based on their abstracts and included if they addressed the correlation between vaginal microbiota and ovarian cancers. A total of 38 articles were identified and summarized. The topics covered have been categorized into the following paragraphs. Supplementary Tables S1–S5 summarize the articles found and selected for this review along with their respective topics covered.

## 3. Results

### 3.1. Vaginal Microbiota: Benefit or Pitfall?

VMB primarily consists of lactic acid-producing *Lactobacillus species*, such as *Lactobacillus crispatus*, *Lactobacillus gasseri*, *Lactobacillus iners*, and *Lactobacillus jensenii*. These beneficial bacteria help maintain a healthy vaginal environment. Disruptions in the VMB, known as dysbiosis, can lead to an overgrowth of pathogenic microorganisms. This imbalance may raise the risk of infections and inflammation, which are linked to the development of various cancers, including ovarian cancer. VMB can influence local immune responses. An imbalance in microbial communities may hinder the immune system’s ability to detect and eliminate cancerous cells, potentially promoting tumor development [5]. Maintaining a healthy VMB could potentially reduce the risk of ovarian cancer [6]. Community status type IV (CST IV), particularly subtypes IV-A (aerobic) and IV-B (anaerobic), represents a dysbiotic vaginal environment characterized by the loss of protective *Lactobacillus* spp. and overgrowth of bacteria as diverse as *Gardnerella vaginalis*, *Atopobium vaginae*, *Prevotella bivia*, and *Fusobacterium nucleatum*. This polymicrobial shift, characteristic of bacterial vaginosis (BV), contributes to carcinogenic processes through the production of short-chain fatty acids (SCFAs) such as acetate and propionate, which can promote tumorigenesis by modulating epithelial integrity, immune signaling, and inflammation. Bacterial enzymes such as sialidases and proteases that degrade mucin further damage the mucosal barrier, facilitating microbial invasion and chronic immune activation. In contrast, butyrate, an SCFA with anti-inflammatory and antineoplastic activity, is typically reduced in this context. These mechanisms reflect broader patterns observed in the gut microbiota, where high microbial diversity, imbalance of SCFAs, and increased intestinal permeability (i.e., leaky gut syndrome) have been implicated in systemic inflammation and oncogenesis, as highlighted in the work of Jacobson et al. [7]. Together, they underscore how vaginal and intestinal microbial ecosystems can shape local and systemic cancer risk through activation of specific microbial pathways. From these microenvironmental dynamics, amino acids (AAs) are increasingly recognized as key mediators at the intersection of host metabolism, microbial metabolism, and oncogenesis. In both the vaginal and intestinal ecosystems, some AAs-such as glutamine, serine, and tryptophan-can be metabolized by dysbiotic microbial communities into bioactive metabolites that promote or suppress tumor processes. In the vaginal microenvironment, bacterial catabolism of AAs under dysbiotic conditions can fuel the production of pro-inflammatory mediators, modulate local pH, and alter epithelial homeostasis. At the same time, AAs can serve as substrates for SCFA production, enhancing the oncogenic or protective role of these metabolites depending on the microbial context. In the intestine, where amino acid fermentation is most extensive, elevated levels of certain microbial byproducts-such as indoles, polyamines, or ammonia-have been linked to epithelial stress, immune modulation, and colorectal cancer. In particular, Jacobson’s work highlights how amino acid-driven dysbiosis and increased intestinal permeability can lead to systemic translocation of microbial metabolites, increasing chronic inflammation and contributing to carcinogenesis at distant sites. These insights suggest that AA flux, shaped by both diet and microbial composition, may act as a metabolic bridge, connecting microbial ecology to tumor biology across mucosal surfaces. The vast array of microorganisms inhabiting mammalian body surfaces has co-evolved with the immune system. This co-evolution has resulted in a balanced relationship in which the immune system tolerates beneficial microbes while remaining vigilant against potential pathogens. The immune system plays a vital role in maintaining homeostasis with resident microbial communities. This balance ensures that the mutualistic relationship existing between the host and its microbiota is preserved, thus preventing dysbiosis that could lead to disease. While many microbes perform functions critical for host physiology, they also pose a threat of invasion, potentially leading to pathologies. The immune system’s ability to distinguish between beneficial and harmful microbes is essential in preventing such breaches.

The microbiota may influence the tumor microenvironment by promoting or inhibiting carcinogenesis. For instance, microbial-driven immune modulation can affect the behavior of tumor-associated macrophages (TAMs) and regulatory T cells (Tregs), which may either suppress or promote tumor growth [8]. Microbial dysbiosis can lead to epithelial barrier dysfunction, immune system dysregulation, genotoxic effects, and inflammation, collectively creating a microenvironment that favors tumor development. Interestingly, certain bacteria, such as Chlamydia trachomatis, can induce epithelial-to-mesenchymal transition in infected cells. This process may result in a loss of adhesion among epithelial cells and a decrease in DNA repair mechanisms, potentially facilitating carcinogenesis. Furthermore, bacterial communities may influence the development, severity, and treatment response of gynecological cancers. These microorganisms might initiate carcinogenesis through direct or indirect mechanisms; however, it is also possible that the tumor microenvironment itself may promote the recruitment and proliferation of anaerobic microorganisms [8,9]. Bacteria and viruses are thought to contribute to about 15% of malignant neoplasms. Oncogenic bacteria and viruses directly affect carcinogenesis by either producing specific toxins that harm host DNA or integrating oncogenes into the host genome [10,11]. A summary of these concepts is available in Supplementary Table S6.

### 3.2. Is There Space for Recommendations?

The International Cancer Microbiome Consortium (ICMC) consensus statement on the role of the human microbiota in carcinogenesis outlines several key recommendations and guidelines to inform future research on how microbiota may influence cancer development. This covers genomic integration (by producing metabolites that interact with host cells, the microbiota may influence the expression of genes related to inflammation, cell cycle regulation, and apoptosis; all of which can result in altered gene expression that promotes tumorigenesis), genotoxicity, inflammation (microbial virulence factors trigger chronic inflammation in host tissue), promoting cellular proliferation that can eventually become dysregulated. When coupled with the failure of apoptosis, this can lead to the development of a malignant phenotype), immunity (while the microbiota may inhibit host immunity, it is believed that, in a healthy state, the interaction between the microbiota and immune system helps maintain an immune “tone” that supports basic anticancer immune surveillance mechanisms), and metabolism (i.e., in the context of colorectal cancer, CRC). The fermentation of dietary fiber by gut bacteria into SCFAs, such as butyrate, is thought to play a crucial role in inhibiting oncogenesis through its anti-inflammatory and antiproliferative effects. Conversely, bacterial metabolism of bile acids and proteins can produce carcinogenic compounds, such as aromatic amines and sulfides, which have been established as mechanisms through which the microbiota may initiate and/or drive carcinogenesis.

The main recommendations of ICMC concentrate on the following topics:

1. **Establishing Causation vs. Association**: It is crucial to distinguish between correlation and causation in microbiota-related cancer research. Simply finding an association between a specific microbiome composition and cancer does not necessarily imply the existence of a causal relationship. Future studies must rigorously assess causality to avoid misinterpretations and confirm whether changes in the microbiota may directly contribute to carcinogenesis [12].

2. **Identifying Microbial Mechanisms of Carcinogenesis**: The consortium advocates for research aimed at identifying the precise molecular mechanisms by which microbial communities influence cancer. Specifically: how do certain microorganisms interact with host cells to promote tumor development? What metabolites or inflammatory pathways are involved in this process? This requires detailed studies of microbial genomics, metabolomics, and host-microbe interactions.

3. **Longitudinal studies**: The consensus calls for large, well-designed longitudinal cohort studies that track patients over time to monitor changes in the microbiome and their potential connections to cancer onset or progression. These studies should include diverse populations to account for variations in genetic, environmental, and lifestyle factors that may influence both the microbiome and cancer risk.

4. **Microbiome Profiling across Cancer Types**: Conduct microbiome profiling across various cancer types to identify common patterns and unique microbial signatures. This research should be comprehensive, considering different body sites and cancer stages (e.g., early vs. late-stage tumors).

5. **Controlled Experimental Studies**: In addition to observational studies, experimental models (e.g., animal models, organoids) should be utilized to investigate how specific microbiota compositions impact cancer-related pathways.

6. **Microbial Therapeutics**: Investigating therapeutic strategies targeting the microbiota includes developing probiotics or prebiotics to modulate the microbiota and reduce cancer risk. Additionally, it involves exploring the use of antibiotics or other microbial therapies to modify the microbial environment and prevent cancer development.

7. **Harmonization of Microbiome Data**: Given the diversity in microbiome research, the consensus highlights the necessity for harmonization across studies. Standardized methods for sample collection, sequencing, data analysis, and reporting are essential for ensuring reproducibility and facilitating comparison of findings across research studies.

8. **Ethical Considerations in Microbiome Research**: Ethical guidelines must be followed when collecting microbiome samples from cancer patients, especially regarding patient consent, data privacy, and the potential implications of microbiome-based diagnostics or treatments. The integration of microbiome data into clinical practice should be approached cautiously, considering patient autonomy and the risk of unintended consequences.

9. **Collaboration Between Different Disciplines to foster a multidisciplinary approach**: Encouraging cooperation among microbiologists, oncologists, immunologists, bioinformaticians, molecular biologists and other related fields, including food scientists, to gain a comprehensive understanding of the microbiota’s role in cancer. Collaborative networks will accelerate the translation of foundational research findings into clinical applications.

10. **Public and Clinical Awareness**: Boost awareness about the microbiome’s potential importance in cancer prevention and therapy. This includes educating clinicians on the microbiome’s role and advocating for its inclusion in cancer diagnostics and treatment planning [13].

### 3.3. Vaginal Microbiota and Ovarian Cancer: Microbial Signatures

Chanyuan et al. investigated the relationship between VMB dysbiosis and ovarian cancer progression. They observed significant alterations in the VMB of ovarian cancer mouse models, indicating a state of dysbiosis. This imbalance was associated with changes in metabolite profiles, particularly those related to amino acid and lysophospholipid metabolism [14]. Administering broad-spectrum antibiotics locally helped reverse the microbiota dysbiosis and suppressed tumor progression in the mouse models, suggesting a potential therapeutic avenue. This highlights a diagnostic potential: given the anatomical challenges of directly assessing ovarian microbiota, the study proposes using vaginal bacteria as non-invasive biomarkers for monitoring ovarian cancer progression. Notably, the presence of the bacterial genus *Burkholderia* demonstrated high diagnostic value, with an area under the curve of 0.8843, indicating strong potential as a biomarker. The results of this study emphasized the significant role of VMB in ovarian cancer progression. The findings suggest that targeting microbiota dysbiosis could serve as both a therapeutic strategy and a diagnostic tool, paving the way for microbe-based interventions in ovarian cancer management. In practice, this research supports the idea of microbiota-targeted interventions, such as probiotics and prebiotics, as novel approaches for managing ovarian cancer [14]. Moreover, changes in the VMB were closely linked to alterations in metabolic pathways in murine models. Healthy controls exhibited a higher abundance of beneficial bacteria, such as *Lactobacillus* spp., which are known to play a protective role by maintaining a balanced vaginal environment. In ovarian cancer models, these protective *Lactobacillus* spp. populations were markedly reduced, contributing to the observed dysbiosis. Lactobacilli help maintain low vaginal pH and produce antimicrobial compounds that inhibit pathogenic bacteria. The depletion of these microbes may create an environment more favorable for tumor-promoting pathogens.

Banerjee et al. identified a unique microbiome signature associated with ovarian cancer [15]. By utilizing a pan-pathogen array (PathoChip) in combination with capture-next generation sequencing, the researchers analyzed ovarian cancer samples alongside both matched and unmatched control samples. Their findings revealed a distinct presence of viral, bacterial, fungal, and parasitic signatures in ovarian cancer cases providing insights that could inform the development of targeted therapeutics for ovarian cancers. Notably, specific viral integration sites within the host genome of tumor samples were identified, suggesting a potential role in the carcinogenic process.

The key species highlighted in their findings were viral signatures:

- Human papillomavirus (HPV)—HPV 6 was found integrated into the host genome in both invasive and borderline tumors, whereas it episomally existed in healthy controls.
- Epstein–Barr virus (EBV) and human cytomegalovirus (CMV) were identified in 50% of ovarian cancer tissues, with an associated elevated risk of ovarian tumor development (OR 8; 95% CI 0.888, 72.10).
- Human herpesvirus 6 and 7 (HHV-6, HHV-7) may modulate the tumor microenvironment. Retroviruses, such as endogenous retroviruses, have been observed in tumor tissues [16].

At first glance, the connection between the papillomavirus family and ovarian cancer may seem unusual, given its well-known link to cervical cancers. Nevertheless, some evidence supports this association with ovarian cancer. The first mechanism involves inhibiting insulin-like growth factor-binding proteins, which increases the availability of free and active insulin-like growth factors that may have mitogenic effects [17]. The second mechanism involves the activation of oncogenic genes, such as SH3RF2, which typically regulate the activity of the p21 protein, involved in cell-cycle arrest [18].

As concerning bacterial signature, it consisted of:

*Fusobacterium nucleatum*—Enriched in ovarian cancer tissues and linked to inflammation and carcinogenesis.

*Propionibacterium acnes*—Associated with immune modulation in the tumor microenvironment.

*Group B Streptococcus*—Detected in ovarian cancer samples, potentially influencing local immune responses.

Shanmughapriya et al. also found Chlamydia trachomatis infection in 80% of cancerous ovarian tissues, with a significantly higher risk reported (odds ratio [OR] 32; 95% confidence interval 3.33, 307.65) of developing infection-related ovarian tumors [15]. Chlamydia trachomatis can ascend from the vagina to the upper reproductive tract, leading to pelvic inflammatory disease (PID). PID has been linked to an increased risk of ovarian cancer [5]. Similarly to Chlamydia trachomatis, all incriminating PID microorganisms share this association. Therefore, Chlamydia trachomatis may promote carcinogenesis by allowing the survival of DNA-damaged host cells or by transferring tubal-derived cells to environments that enhance ovarian growth [14].

Also, fungal signatures were identified, including *Candida albicans*, which influence inflammatory responses and tumor progression, along with *Aspergillus* spp.

These microbial signatures indicate complex interactions between the oncobiome and the tumor microenvironment, which may influence cancer initiation, progression, and immune modulation [18].

In particular, a potential role has been examined in the context of recidivism. Jacobson et al. found that patients with shorter recurrence times exhibited decreased microbial diversity in the gut. Specific microbial taxa, such as a reduction in Ruminococcaceae and an increase in Proteobacteria, were associated with faster recurrence. They also noted a loss of dominance by *Lactobacillus* spp. in the VMB of patients with shorter recurrence intervals, along with the presence of non-*Lactobacillus* spp., such as *Escherichia coli*, linked to chemoresistance and worse clinical outcomes [7].

### 3.4. The Reported Populations of Microorganisms: A Closer Look

VMB is classified into five community state types (CSTs): CST I—*Lactobacillus crispatus*; CST II—*Lactobacillus gasseri*; CST III—*Lactobacillus iners*; CST IV—Characterized by low presence of Lactobacilli and high microbial diversity; CST V—*Lactobacillus jensenii.* The majority fall into CSTs I through III, which are mainly composed of *Lactobacillus* spp., including *Lactobacillus crispatus*, *Lactobacillus gasseri*, and *Lactobacillus iners*. A smaller subset belongs to CST IV, which includes various strictly and facultative anaerobic bacteria or a microbiota more susceptible to bacterial vaginosis, characterized by higher levels of *Atopobium vaginae*, *Gardnerella vaginalis*, *Prevotella bivia*, and *Parvimonas* micra [19].

The composition of the VMB also appears to vary depending on the female populations of different ethnic groups. For example, a reduced prevalence of Lactobacilli has been observed in black women, with a predominance of *Atopobium* and *Clostridiales* [20]. Indirectly, one could assume a higher incidence of correlation with the onset of ovarian neoplasia, but this remains an assumption that needs to be investigated from scratch.

VMB with fewer than 50% dominance of *Lactobacillus* spp. has been defined as Community Type O. This is more prevalent among ovarian cancer patients and individuals with BRCA1 mutations.

A recent case–control study investigated the connection between cervicovaginal microbiota and ovarian cancer by analyzing three distinct groups: (i) women diagnosed with epithelial ovarian cancer prior to treatment; (ii) individuals with a BRCA1 mutation who do not have ovarian cancer, and (iii) a control group consisting of healthy individuals as well as those bearing benign gynecological conditions.

One of the key findings was that, among the 85 participants under the age of 50, the majority (81%) were premenopausal. Interestingly, women with ovarian cancer were significantly more likely to exhibit a Lactobacillus-depleted microbiota (defined as Community Type O) compared to age-matched controls. After adjusting for key variables, the study found that ovarian cancer patients had an odds ratio of 2.80 (95% CI: 1.17–6.94) for developing this type of microbiota, suggesting a potential association between dysbiosis and ovarian cancer.

The study also found that BRCA1 mutation carriers under 50 were more prone to have a Community Type O microbiota compared to those without the mutation. This observation raises intriguing questions about the potential genetic influence on microbiota composition, as the human genome plays a role in regulating tissue-associated microbial communities. It is possible that BRCA1 loss-of-function mutations not only contribute to cancer susceptibility but also may affect the gut and cervicovaginal microbiota, further complicating the understanding of ovarian cancer development.

Beyond genetic predisposition, external factors were also examined regarding microbiota composition and cancer risk. The study observed that smoking adversely affects the presence of *Lactobacillus* spp., potentially fostering a more dysbiotic and inflammatory environment that could contribute to carcinogenesis. Conversely, estrogen-based hormonal contraception and hormone replacement therapy (HRT) appeared to enhance *Lactobacillus* spp. dominance, which may have protective effects against ovarian cancer.

However, one of the biggest challenges in interpreting these findings is the possibility of reverse causation. Instead of dysbiosis having a causal role in the development of ovarian cancer, it is also possible that the cancer itself may alter the tumor microenvironment, leading to changes in microbiota composition. This raises the question of whether microbial imbalances contribute to cancer initiation or if they are merely a consequence of the disease process.

Overall, while the study highlights fascinating connections between ovarian cancer, the vaginal microbiota, and BRCA1 mutations, further research is necessary to determine whether changes in microbiota result from the disease or contribute to its onset. Gaining a deeper understanding of this relationship could open up new avenues for microbiota-targeted therapies, early detection biomarkers, and even preventive strategies for individuals at risk [21].

Regardless of their menopausal state, ovarian cancer patients exhibit microbiota similar to that of healthy postmenopausal women, characterized by increased microbial diversity and elevated levels of *Propionibacterium* spp. and *Corynebacterium* spp. These changes can even be observed in early-stage ovarian cancers [22].

A study highlighted that a significant reduction in *Lactococcus* spp. levels in ovarian cancer tissue could serve as a potential biomarker for the early detection of the disease. However, the limited research specifically focusing on the ovarian microbiota leaves the role of microbes within the ovaries ambiguous [23].

*Clostridium* cluster XIVa, a group of bacteria that produce high levels of butyrate, was negatively associated with ovarian cancer tumor scores. The inhibitory effects of butyrate on cancer cell growth may rely on its role in promoting regulatory T cells (Tregs). Escherichia coli lipopolysaccharides trigger inflammatory responses in ovarian tumor cells, promoting growth and chemoresistance via the inflammatory TLR-4-MyD88 pathway. TLR signaling, a known driver of inflammatory responses, may facilitate ovarian cancer progression. Intratumoral microbes may activate inflammation-induced hedgehog signaling pathways, contributing to the progression of ovarian cancer. Vaginal microbes may influence the upper genital tract through microbial ascension, which refers to bacteria moving from the vagina to the ovaries through structural continuity in the reproductive tract, or through inflammatory connections, in which chronic conditions, such as pelvic inflammatory disease and endometriosis, might predispose the development of ovarian cancer through sustained inflammation and genomic instability.

Chan et al. investigated the presence of *Mycoplasma* spp. DNA in ovarian cancer tissues compared to vaginal tissues, which served as control non-malignant samples. Utilizing sensitive PCR–ELISA methods, the researchers detected *Mycoplasma* spp. DNA in 59.3% of the analyzed ovarian cancer samples. By including vaginal tissues as controls, the researchers aimed to determine whether the presence of *Mycoplasma* spp. DNA was specific to ovarian cancer or part of the natural microbial composition of the female reproductive tract. The significantly higher prevalence of *Mycoplasma* spp. DNA, in ovarian cancer tissues compared to vaginal tissues, suggests a potential association with ovarian carcinogenesis, rather than a generalized presence in the reproductive tract. The study hypothesizes that *Mycoplasma* spp. could contribute to cancer development through mechanisms such as chronic inflammation, immune evasion by the tumor, and induction of genomic instability in host cells. These findings underline the need for further research to clarify whether *Mycoplasma* spp. act as a cofactor in cancer development or merely reflect an opportunistic infection in tumor environments [24]. The etiopathogenetic link appears to be such that Cazzaniga et al. have already summarized the oncogenic mechanisms of ovarian cancer in a descriptive scheme, highlighting the decrease in the proportion of lactobacilli and the increase in the bacterial populations of *Proteobacteria* and *Firmicutes phyla*. This is accompanied by a proportional rise in anaerobic bacteria, as well as infections with *Mycoplasma* spp. or *Chlamydia* spp., and retroviruses, including Herpesvirus, HPV, and CMV. These factors, along with genetic and environmental influences, contribute to the transformation of ovarian cells into cancerous cells.

BV is a dysbiotic condition of the vaginal microbiota, primarily initiated by *Gardnerella vaginalis* (GV), which disrupts the protective dominance of lactobacilli. GV serves as a key initiator, forming biofilms that facilitate the growth of a polymicrobial community comprising *Atopobium vaginae*, *Prevotella bivia*, *Mobiluncus* spp., and *Fusobacterium nucleatum*. These organisms work synergistically to destabilize the vaginal ecosystem, leading to the characteristic symptoms of BV and a metabolically altered environment. Recent studies have begun to uncover how specific cervical metabolites produced by these microbes-such as acetate and propionate-may act as oncogenic promoters, while others, such as butyrate, may play antineoplastic and immunomodulatory roles. These new insights into the metabolic profile of BV not only deepen our understanding of the dynamics of infection but also lay the groundwork for exploring the role of AAs in shaping the local microenvironment, potentially serving as key metabolic signals or modulators in health and disease.

*Gardnerella vaginalis* is frequently associated with bacterial vaginosis (BV) and has been linked to a higher risk of gynecological cancers. It forms biofilms that may protect cancer cells from chemotherapy drugs, potentially reducing treatment effectiveness. The inflammation caused by *Gardnerella vaginalis* may create a microenvironment that encourages cancer progression and chemoresistance. *Atopobium vaginae* often coexists with *Gardnerella vaginalis* in cases of bacterial vaginosis. It contributes to a dysbiotic VMB and has been associated with HPV persistence and cervical cancer. Some studies indicate that its presence may diminish the effectiveness of chemotherapy by promoting chronic inflammation and fostering a tumor-friendly microenvironment. *Prevotella* spp. have been connected to immune suppression, which compromises the efficacy of immunotherapies in gynecological cancers. *Escherichia coli* in the vaginal or gut microbiome can affect drug metabolism, resulting in increased toxicity or decreased effectiveness of chemotherapy agents. In some cases, chemotherapy-induced gut dysbiosis can lead to the overgrowth of harmful Escherichia coli strains, exacerbating inflammation and side effects [25].

Finally, Zhou et al. investigated the differences in microbiota between ovarian cancer tissues and normal distal fallopian tube tissues. Utilizing 16S rRNA high-throughput sequencing, the researchers analyzed 25 samples from each tissue type. Notably, there was an increased ratio of *Proteobacteria* to *Firmicutes phyla* in ovarian cancer samples, suggesting a potential link between changes in microbial composition and the development of ovarian cancer. Additionally, transcriptome sequencing revealed distinct transcriptional profiles between the two tissue types, with modulation observed in 84 inflammation- or immune-associated genes. The study hypothesizes that alterations in microbial composition may influence the initiation and progression of ovarian cancer by affecting the local immune microenvironment of the fallopian tubes [26].

### 3.5. Role of Amino Acids in Tumor Microenvironment

Vaginal dysbiosis has been found to alter the metabolism of AAs in a way that complicates the process of carcinogenesis. Abnormal levels of specific AAs have been detected, which may contribute to the tumor microenvironment [27,28,29].

For instance, glutamine is a critical nutrient for tumor cells, fueling the tricarboxylic acid (TCA) cycle and promoting growth and survival. Indeed, increased levels of glutamine were detected in the tumor environment, and glutamine was demonstrated to supporting proliferation of ovarian cancer cells [30]. Abnormal tryptophan metabolism was also noted, often linked to immune evasion in tumors. Tryptophan catabolism can result in production of kynurenine, which suppresses T-cell activity [31] and fosters a tumor-permissive immune environment through the recruitment and polarization of regulatory T cells [32].

Higher levels of arginine were found, potentially promoting tumor proliferation and angiogenesis (the formation of new blood vessels). Indeed, arginine can be converted into ornithine and urea by the enzymes arginases or can be used for nitric oxide production. Arginine metabolism can have dual effects, supporting both tumor progression and immune responses [33]. Moreover, dysregulation of arginine and ornithine availability may boost polyamine production, which aids in DNA stabilization and cancer cell proliferation [33].

Lower levels of cysteine have been reported, which may decrease glutathione production (a key antioxidant) and increase oxidative stress in the tumor microenvironment. Serine depletion has also been noted, which could hinder normal cellular functions as it is diverted to cancer-specific biosynthetic pathways. Reduced glycine levels may suggest altered one-carbon metabolism, often reprogrammed in cancer cells to facilitate nucleotide synthesis. Abnormal levels of branched-chain AAs (BCAAs), such as leucine, isoleucine, and valine, have been linked to changes in signaling pathways that promote tumor growth. Disruptions in methionine metabolism may lead to epigenetic modifications (e.g., DNA methylation) that drive oncogenic gene expression [34]. The altered levels of these AAs, influenced by VMB dysbiosis, play a critical role in shaping the tumor microenvironment [35,36]. They contribute to processes such as immune evasion, oxidative stress, angiogenesis, and the proliferation of cancer cells. These findings suggest that targeting amino acid metabolism may offer therapeutic potential for managing ovarian cancer [14]. Supplementary Figure S1 summarizes these features in a heatmap.

Glutamine metabolism is well supported by preclinical and clinical tumor metabolomics studies: glutamine fuels the TCA cycle and nucleotide synthesis, consistently acting in a *pro-tumor* direction by sustaining proliferation and survival [30]. Tryptophan catabolism via the kynurenine pathway is also supported by strong preclinical evidence and correlative clinical data, fostering pro-tumor immune evasion by suppressing effector T cells and promoting regulatory T cell polarization [31,32]. Arginine metabolism shows mixed effects: preclinical studies demonstrate pro-tumor roles (angiogenesis, polyamine production, DNA stabilization), but arginine can also support anti-tumor immunity via nitric oxide generation, highlighting context-dependent directionality [33]. Cysteine and serine depletion are mostly preclinical findings that reduce antioxidant capacity (glutathione) and redirect carbon flux into cancer-specific biosynthesis, again acting in a pro-tumor fashion. Glycine and methionine alterations are supported by tumor metabolomic and epigenetic studies, where one-carbon metabolism and DNA methylation changes consistently promote oncogenic programming. Branched-chain amino acids (BCAAs), though less studied in ovarian cancer specifically, show preclinical evidence of pro-tumor signaling activation (e.g., mTOR).

### 3.6. Gut–Vaginal Microbiota Axis: Is There a Real Connection?

The “gut microbiota” is the collective term for the trillions of microorganisms—primarily bacteria, but also viruses, fungi, and archaea—that reside in the human digestive tract, particularly in the intestines [34]. These microbes form a complex and dynamic ecosystem that plays an essential role in maintaining human health. They help break down dietary fiber and complex carbohydrates that the body cannot digest on its own, producing SCFAs and other metabolites that promote gut health [35]. The microbiota also contributes to the synthesis of certain vitamins (such as B12 and K), regulates immune function, and helps prevent colonization by harmful pathogens through a mechanism known as competitive exclusion [10,36,37]. Most of the gut microbiota consists of bacteria, with two major phyla—*Firmicutes* and *Bacteroidetes*—dominating in healthy individuals. These microbes also produce particularly SCFAs that support gut lining integrity and reduce inflammation. The composition of an individual’s gut microbiota is shaped by factors such as diet, lifestyle, environment, age, and even mode of birth (vaginal or cesarean). Imbalances in this microbial community, known as “dysbiosis,” have been linked to a range of conditions, including gastrointestinal disorders such as inflammatory bowel disease, metabolic diseases such as obesity and type 2 diabetes, and even neurological conditions such as depression and anxiety.

Beyond its roles in metabolism and immunity, emerging research has uncovered a link between the gut microbiota and oncogenesis. Certain microbial imbalances may contribute to chronic inflammation, DNA damage, or immune suppression, potentially promoting the development of cancers, particularly in the colon [38]. Understanding and nurturing the gut microbiota is increasingly recognized as a key aspect of personalized medicine and preventive healthcare [39].

Concerning ovarian cancer, the gut microbiota has been considered particularly significant [40], as often the disease firstly presents through gastrointestinal symptoms like abdominal pain and bloating. Evidence from preclinical models suggests that gut microbiota play a crucial role in regulating inflammatory responses necessary for both immunotherapy and chemotherapy. This indicates that manipulating specific bacterial populations in the gut may enhance the effectiveness of cancer treatment, presenting a potential therapeutic target [40].

In light of the connection existing between vaginal and intestinal microbiota and ovarian cancer, it has also been revealed that cancer-related cachexia results from increased intestinal permeability due to microbial pathogens or inflammation, potentially triggering a systemic immune response. Chemotherapy may exacerbate this process by further increasing intestinal permeability, leading to chronic inflammation, metabolic dysfunction, and malnutrition. Therefore, maintaining gut barrier integrity could help mitigate the symptoms of cachexia. The “GI microbiota–muscle axis” has also become a concrete concept, suggesting that gut bacteria can impact muscle mass both positively and negatively. While the microbiota contributes to amino acid availability, which is essential for muscle maintenance, it can also activate TLRs, triggering NF-κB signaling, promoting inflammation and muscle wasting. This indicates that gut bacteria play a crucial role in the development of cachexia. An additional study in a leukemia mouse model found that cachexia was associated with a reduced abundance of *Lactobacillus* spp. in the gut. Supplementing with *Lactobacillus reuteri* and *Lactobacillus gasseri* decreased inflammatory cytokines and increased muscle mass, indicating that gut microbiota alterations contribute to cachexia. While these findings are promising, validation in human studies is needed by comparing the microbiota composition in healthy individuals versus cachectic ovarian cancer patients [41,42,43].

The study by Morikawa et al. found that premenopausal ovarian cancer patients exhibit a cervicovaginal microbiota shift resembling that of healthy postmenopausal women, who are characterized by reduced dominance of *Lactobacillus* spp. and increased microbial diversity. Similar common differences were observed across various histotypes, such as high-grade serous ovarian cancers and clear cell types [44].

Similarly, from the gut microbiota studies, we derived insights suggesting that compounds secreted by Enterococcus faecium may possess potential anticancer properties against ovarian cancer. The effects were observed through the inhibition of cancer cell growth, as *Enterococcus faecium* significantly reduced Caov-4 cell viability in a dose- and time-dependent manner. Additionally, induction of apoptosis was observed, with treated cells exhibiting DNA fragmentation, nuclear condensation, and increased apoptotic markers, confirming programmed cell death. Gene expression changes included the upregulation of pro-apoptotic genes (BAX, PTEN) and the downregulation of anti-apoptotic genes (BCL2, AKT1), which promoted cell death [45].

In recent years, the concept of modulating the gut microbiota as a means to prevent or treat cancer has evolved from theoretical to highly promising. Researchers are just discovering how deeply the microbiota influences not only our digestive and immune systems, but also how our bodies respond to cancer and its treatments. This complex community of microorganisms, particularly in the gut, can impact a wide range of processes, including inflammation, immune signaling, and the effectiveness of chemotherapy and immunotherapy.

One of the key strategies under exploration involves dietary and microbial interventions. For example, prebiotics—which are non-digestible plant fibers—serve as nourishment for beneficial bacteria. By feeding genera like *Bifidobacterium* and *Lactobacillus*, prebiotics can help shift the microbial balance toward a more protective state, reducing pro-inflammatory or carcinogenic bacterial populations [46].

Likewise, probiotics, which are live beneficial bacteria, have shown promise in modulating the immune response, strengthening the intestinal barrier, and even producing substances that can inhibit the growth of cancer cells. Certain strains, such as Lactobacillus rhamnosus, may also aid in managing the side effects of cancer treatments, like chemotherapy-induced gut damage [47].

Then, we have postbiotics, which are not live organisms but beneficial compounds produced by microbes—like SCFAs, such as butyrate. Butyrate is particularly interesting because it helps maintain the integrity of the gut lining and can even induce cancer cell death, especially in the colon.

A combination of prebiotics and probiotics, known as symbiotics, aims to maximize the benefits of both. These are currently being investigated for their potential role in enhancing gut health and supporting immune function during cancer therapy [48].

Beyond dietary approaches, more advanced clinical interventions are gaining traction. Fecal microbiota transplantation (FMT)—the transfer of stool from a healthy donor to a patient—has been successfully used to treat gut infections and is now under investigation in oncology. Early studies indicate that FMT may enhance the effectiveness of immune checkpoint inhibitors in cancers like melanoma by reintroducing beneficial bacteria that help activate the body’s immune defenses.

Another experimental yet intriguing technique is vaginal microbiota transplantation (VMT). While still in its early stages, VMT is currently being studied in women at risk for gynecological cancers, particularly those linked to HPV infection. The aim is to restore a healthy, *Lactobacillus* spp.-rich vaginal environment, which may help reduce viral persistence and inflammation—two factors linked to the development of cervical cancer [49]. Together, these approaches reflect a broader shift in how we view cancer therapy—not only targeting the tumor but also supporting the body’s internal environment to fight back more effectively. As our understanding of the microbiota improves, it is becoming evident that modulating these microscopic allies could play a crucial role in more personalized and holistic cancer care.

## 4. Discussion

Most links between the vaginal microbiota and ovarian cancer are correlational rather than causal. The idea that microbes ascend from the vagina to the ovaries is proposed but still not definitely demonstrated. Confounding factors like age, hormones, and BRCA status complicate interpretation, and ovarian cancer itself could secondarily alter the microbiota. Mechanistic studies and longitudinal data are still needed to clarify whether dysbiosis contributes to carcinogenesis or is merely a consequence.

### 4.1. Current Evidence and Limitations

Emerging research on the role of vaginal microbiota in ovarian cancer suggests that microbial imbalances—dysbiosis—may be associated with the development of the disease but could also influence treatment outcomes. However, evidence remains inconclusive. Capozzi et al. found no significant difference in the prevalence of genital dysbiosis between ovarian cancer patients and healthy controls, despite prior studies linking dysbiosis to cervical and endometrial cancers. [50].

These findings highlight the need for cautious interpretation, as most studies to date are correlational rather than causal.

### 4.2. Diagnostic Potential

Non-invasive vaginal microbiota screening has been proposed as an effective tool for early-stage ovarian cancer detection. Dysbiosis may trigger chronic inflammation and metabolic alterations that promote tumorigenesis, while specific microbial metabolites could influence hormonal and metabolic pathways relevant to cancer progression. Identifying microbial signatures specific to ovarian cancer could therefore improve risk stratification. This microbiome screening could complement existing diagnostic methods, offering a more comprehensive view of an individual’s risk. Furthermore, combining the microbiome profile with other diagnostic biomarkers, such as mutational and methylation analyses, could enhance the accuracy of ovarian cancer detection.

Restoring microbiota balance through probiotic treatments (e.g., *Lactobacillus* spp. supplements) may reduce inflammation and improve immune responses, making the body more resilient to cancer therapies such as chemotherapy and immunotherapy. Additionally, antibiotic stewardship is vital in preventing dysbiosis during cancer treatment. While antibiotics are essential for treating infections, they can disrupt the delicate balance of the microbiota, potentially leading to conditions that worsen cancer development. Therefore, preventing such disruptions through mindful antibiotic use is crucial for maintaining a healthy microbiota during treatment.

There is need for additional experimental approaches, in which microbiota transplants could be explored to restore a beneficial microbiota, either before or during cancer therapy, thereby helping to enhance treatment efficacy and minimize side effects (Supplementary Figure S2). These innovative treatments might be integrated into personalized cancer care, where therapies are customized to the individual based on their microbiome composition.

Looking toward the future, microbiota profiling could become a cornerstone of personalized gynecologic cancer therapy. Standardized protocols for implementing microbiota-modulating interventions—such as determining optimal probiotic strains or dietary recommendations—are needed to incorporate these approaches into clinical practice. Additionally, further clinical trials will be necessary to assess the safety and effectiveness of these microbiota-based interventions in gynecologic cancer patients. Van Bommel et al. demonstrate that mutational testing of cervicovaginal and endometrial samples has limited sensitivity, underscoring the need for complementary approaches that include microbiome-based strategies [51].

Furthermore, about potential relationship between BRCA1/2 genes and ovarian cancer, we know that BRCA1/2 mutations may directly reshape the vaginal environment by altering epithelial turnover, immune signaling, hormone regulation, and mucosal barrier integrity. These changes disrupt conditions that normally support Lactobacillus dominance, leading to dysbiosis. Such microbiota shifts can amplify inflammation and genotoxic stress, creating a feedback loop that may further increase ovarian cancer risk [52].

Reverse causation could be assumed actually; therefore, the need for prospective studies is desirable.

### 4.3. Population and Racial Differences

Racial and population-level differences in microbiota composition warrant closer attention. For example, African American women show distinct vaginal metabolomic profiles compared to White women, suggesting that microbiota-related cancer risks may differ across populations. Broader representation in future studies will be critical to ensure findings are generalizable beyond Western cohorts [53]. Other potential confounding factors—such as sexual activity, antibiotic use, and hormonal status—should be considered in the correlation definition between these two entities.

### 4.4. Therapeutic Implications

Microbiota-modulating interventions represent promising strategies to support cancer therapy [54,55].

Restoring *Lactobacillus* spp. balance through probiotic supplementation may reduce inflammation, strengthen immune responses, and improve outcomes from chemotherapy and immunotherapy. Equally important is antibiotic stewardship, since unnecessary antibiotic use can disrupt the microbiota and potentially worsen cancer-related outcomes. More experimental approaches, such as microbiota transplantation, are also being investigated to restore beneficial communities before or during treatment, potentially improving efficacy and minimizing side effects.

### 4.5. Future Directions

Ovarian cancer is often marked by loss of vaginal Lactobacillus dominance, increased microbial diversity, and enrichment of anaerobes such as Gardnerella and Prevotella, patterns that can distinguish it from other gynecologic conditions. However this should be confirmed on high level of evidence studies. Rather than relying on a single species, the most promising approach is a multimarker microbial signature combined with host and clinical data. To standardize this for screening, protocols must unify sample collection (e.g., vaginal swabs), storage and sequencing methods, and apply reproducible analytic pipelines. Validated classifiers should then be tested across diverse populations in prospective studies, with clear thresholds and quality controls to ensure reliability in clinical programs.

Furthermore, in a prospective view, microbiota-modulating strategies in ovarian cancer should be personalized by first profiling each patient’s gut and vaginal communities and then tailoring interventions such as Lactobacillus-based probiotics, prebiotic or dietary support or precision microbiota transplants to correct specific dysbiosis patterns.

Future work should aim to identify microbial signatures most strongly associated with ovarian cancer risk and treatment response. Developing standardized protocols for probiotic strains, dietary recommendations, and other microbiota-modulating interventions will be essential for translation into clinical practice. Well-designed clinical trials are also necessary to confirm the safety and efficacy of these strategies and to clarify whether observed microbial shifts are causes or consequences of ovarian cancer.

Although the current evidence does not establish a causal link between vaginal dysbiosis and ovarian cancer, microbial imbalance remains an important factor that deserves attention. Incorporating microbiota-focused approaches into prevention, diagnostics, and therapy holds promise for improving patient outcomes. With continued research, microbiota-based strategies may become key components of personalized gynecologic cancer care.

Our intent was simply to summarize the evidence. An additional examination was conducted on amino acid metabolism and the integration of the gut–vaginal axis, and therapeutic implications were discussed, particularly to provide gynecologists with some food for thought from current research for their daily clinical practice.

It is vital to emphasize that vaginal dysbiosis is not a minor issue and deserves our attention; it should be acknowledged and treated appropriately, as it can be regarded as a means of cancer prevention. We therefore suggest a summary of key points to consider in this regard. Future studies will show how accurate or inaccurate our suggestions have been to date.

## 5. Conclusions

Restoring a healthy vaginal and uterine microbiota may help reduce the risk of gynecologic cancer. Possible interventions include probiotics (e.g., *Lactobacillus* spp. supplements) to maintain a protective microbiota, prebiotics to encourage the growth of beneficial bacteria, and dietary interventions (e.g., fiber-rich diets) to support a healthy microbiota.

Moreover, the composition of the microbiota could affect the efficacy of chemotherapy and immunotherapy. Therefore, possible approaches to enhance treatment outcomes will be explored in the future, such as probiotic supplementation to reduce inflammation and improve immune response, antibiotic stewardship to prevent dysbiosis during cancer treatment, and microbiota transplants (an experimental approach) to restore a beneficial microbiota before or during therapy.

## Figures and Tables

**Table 1:** Strings of research.

Research	Findings
((“Carcinoma, Ovarian Epithelial”[Mesh]) AND “Tumor Microenvironment”[Mesh])	286
((“Tumor Microenvironment/drug effects”[Mesh] OR “Tumor Microenvironment/genetics”[Mesh] OR “Tumor Microenvironment/immunology”[Mesh]) AND “Ovarian Neoplasms”[Mesh]) AND “Organoids”[Mesh]	2
(“Tumor Microenvironment/drug effects”[Mesh] OR “Tumor Microenvironment/genetics”[Mesh] OR “Tumor Microenvironment/immunology”[Mesh]) AND “Ovarian Neoplasms”[Mesh]:	438
(((“Tumor Microenvironment/drug effects”[Mesh] OR “Tumor Microenvironment/genetics”[Mesh] OR “Tumor Microenvironment/immunology”[Mesh]) AND “Ovarian Neoplasms”[Mesh]) AND “Recurrence”[Mesh] AND “Organoids”[Mesh]) and also without “AND Organoids”	No results

## Data Availability

No new data were created or analyzed in this study. Data sharing is not applicable to this article.

## Supplementary Materials

The following supporting information can be downloaded at: https://www.mdpi.com/article/10.3390/cells14201590/s1, Figure S1: Amino Acid Alterations and Oncogenic Mechanisms; Figure S2: Microbiota-related mechanisms across gynecologic cancers; Table S1: Ovarian Cancer & Microbiome; Table S2: Vaginal Microbiota & Gynecologic Cancers; Table S3. Microbiome & Cancer Progression; Table S4. Experimental Models & Diagnostic Approaches; Table S5. Microbiome & Cancer Therapy; Table S6: A summary of the microbiota’s concepts.
