# Supplementary material for: The Relationship Between the Vaginal Microbiota and the Ovarian Cancer Microenvironment: A Journey from Ideas to Insights
Source: Cells. 2025 Oct 13;14(20):1590. doi: 10.3390/cells14201590 (PMC12563121; doi:10.3390/cells14201590)
Supplement: Supplementary file 1 [file cells-14-01590-s001.zip › cells-3866237-supplementary.pdf]

Supplementary Figure S1.

Amino Acid Alterations and Oncogenic Mechanisms

Alteration in Tumor Microenvironment	Proposed Oncogenic Mechanism
Glutamine	Fuels the TCA cycle, supports tumor growth and survival
Tryptophan	Promotes immune evasion by suppressing T-cell activity and recruiting regulatory T cells
Arginine	Stimulates proliferation and angiogenesis
Cysteine	Enhances proliferation and angiogenesis through redox balance
Serine	Reduces glutathione, increases oxidative stress, supports nucleotide synthesis
Glycine	Diverted to cancer-specific biosynthetic pathways (e.g., purine synthesis)
Glycine (via one-carbon metabolism)	Alters one-carbon metabolism, supporting nucleotide synthesis and methylation reactions
Methionine	Modulates signaling pathways and drives epigenetic modifications that promote oncogenic gene expression

Supplementary Figure S2.

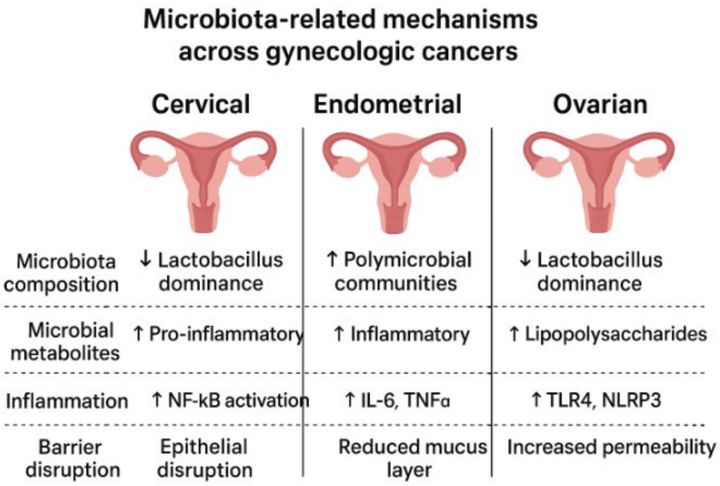

Supplementary Table S1

Ovarian Cancer & Microbiome

Title	Year	First Author	Main topic/findings
The ovarian cancer oncobiome	2017	Sagarika Banerjee	Identified a unique microbiome signature in ovarian cancers, providing insights for targeted therapeutics.
Exploring the Relationship between Ovarian Cancer and Genital Microbiota: A Systematic Review and Meta-Analysis	2021	Vito Andrea Capozzi	Systematically reviewed and analyzed the relationship between ovarian cancer and genital microbiota
Association between the cervico-VMB, BRCA1 mutation status, and risk of ovarian cancer: a case-control study	2021	Nuno R. Nené	Investigated the association between cervicoVMB composition, BRCA1 mutation status, and ovarian cancer risk.

The role of the microbiome in ovarian cancer: mechanistic insights into oncobiosis and bacterial metabolite signaling	2021	Adrienn Sipos	Explored the mechanistic role of the microbiome in ovarian cancer development and progression
Potential Role of Vaginal Microbiota in Ovarian Cancer Carcinogenesis, Progression, and Treatment	2021	Xiumiao Zhao	Investigated the potential role of vaginal microbiota in ovarian cancer development, progression, and treatment response.
Association between vaginal microbiota and the progression of ovarian cancer	2021	Chanyuan Li	Analyzed the relationship between vaginal microbiota composition and ovarian cancer progression.
Altered cervicovaginal microbiota in premenopausal ovarian cancer patients	2021	Asuka Morikawa	Studied changes in cervicovaginal microbiota in premenopausal ovarian cancer patients.
Shifts in gut and VMBs are associated with cancer recurrence time in women with ovarian cancer	2022	David Jacobson	Studied how changes in gut and VMBs correlate with cancer recurrence in ovarian cancer patients.
Ovarian Microbiota, Ovarian Cancer and the Underestimated Role of HPV	2022	Massimiliano Cazzaniga	Highlighted the role of ovarian microbiota in ovarian cancer and discussed the underestimated involvement of HPV.
Prevalence of Mycoplasma Conserved DNA in Malignant Ovarian Cancer Detected Using Sensitive PCR–ELISA	2021	Philip J. Chan	Investigated the prevalence of Mycoplasma DNA in ovarian cancer using PCR-ELISA techniques.
The biodiversity composition of microbiome in ovarian carcinoma patients	2018	Bo Zhou	Analyzed the biodiversity of microbiome composition in ovarian carcinoma patients and its potential impact on cancer progression.

Supplementary Table S2

Vaginal Microbiota & Gynecologic Cancers

**Title**	**Year**	**First author**	**Main topic/findings**
The VMB: IV. The Role of VMB in Reproduction and in Gynecologic Cancers	2022	Gaia Ventolini	Focused on the role of VMB in reproduction and its association with gynecologic malignancies.

Vaginal microbiota and gynecological cancers: a complex and evolving relationship	2021	Kasra Javadi	Discussed the intricate relationship between vaginal microbiota and gynecological cancers.
Gynecological Cancers and Microbiota Dynamics: Insights into Pathogenesis and Therapy	2021	Giovanna Cocomazzi	Provided insights into how microbiota dynamics influence the pathogenesis and treatment of gynecological cancers.
VMBs and ovarian cancer: a review	2021	Jinyun Xu	Reviewed the existing literature on VMBs and their potential role in ovarian cancer development.
Viral and bacterial aetiologies of epithelial ovarian cancer	2021	S. Shanmughapriya	Investigated viral and bacterial causes of epithelial ovarian cancer, suggesting potential infectious etiologies.
The vaginal and gastrointestinal microbiomes in gynecologic cancers: A review of applications in etiology, symptoms, and treatment	2021	Dana Chase	Reviewed the role of vaginal and gastrointestinal microbiomes in gynecologic cancers, discussing their potential impact on cancer etiology, symptoms, and treatment strategies.
The Female Reproductive Tract Microbiome—Implications for Gynecologic Cancers and Personalized Medicine	2021	Anthony E. Rizzo	Explored how the reproductive tract microbiome may influence gynecologic cancers and its potential applications in personalized medicine.
Association between vaginal microbiota and the progression of ovarian cancer	2021	Chanyuan Li	Analyzed the relationship between vaginal microbiota composition and ovarian cancer progression.

Supplementary Table S3

Microbiome & Cancer Progression

**Title**	**Year**	**First author**	**Main topic/findings**
Case for a role of the microbiome in gynecologic cancers: Clinician's perspective	2021	Ismail Mert	Presented a clinician's perspective on the potential role of the microbiome in gynecologic cancers.
The microbiome and gynecological cancer development, prevention, and therapy	2021	Paweł Łaniewski	Discussed the role of the microbiome in the development, prevention, and treatment of gynecological cancers.
Interactions between the microbiota and the immune system	2021	Lora V. Hooper	Reviewed the interplay between the microbiota and the immune system,

			highlighting implications for health and disease.
International Cancer Microbiome Consortium consensus statement on the role of the human microbiome in carcinogenesis	2021	Alasdair J. Scott	Summarized expert consensus on the role of the human microbiome in cancer development and its implications for research and treatment.
Microbiome science needs a healthy dose of skepticism	2021	Not specified	Critically examined the claims regarding microbiome science, emphasizing the need for skepticism and rigorous study design.

Supplementary Table S4

Experimental Models & Diagnostic Approaches

Title	Year	First Author	Main topic/findings
Epithelial Ovarian Cancer Experimental Models	2021	E. Lengyel	Discussed various experimental models used in the study of epithelial ovarian cancer, including in vitro and in vivo systems.
Diagnostic accuracy of mutational analysis along the Müllerian tract to detect ovarian cancer	2021	Majke H.D. van Bommel	Investigated the diagnostic accuracy of mutational analysis in the Müllerian tract for early detection of ovarian cancer.
Human Epididymis Protein 4 and Secretory Leukocyte Protease Inhibitor in Vaginal Fluid: Relation to Vaginal Components and Bacterial Composition	2021	Theofano Orfanelli	Explored the relationship between Human Epididymis Protein 4, vaginal fluid components, and bacterial composition in the context of ovarian cancer.
Synchronous Occurrence of Brucellosis and Ovarian Cancer – A Case Report	2021	Maha Mohamed Emara	Reported a rare case of simultaneous brucellosis infection and ovarian cancer, discussing possible immunological interactions.

Supplementary Table S5

Microbiome & Cancer Therapy

Title	Year	First Author	Main topic/findings
Anticancer Effect of Enterococcus faecium, Isolated from Vaginal Fluid, on Ovarian Cancer Cells	2021	Soraya Pourmollaei	Investigated the potential anticancer effects of Enterococcus faecium, a VMB component, against ovarian cancer cells.
Modulatory Role of Vaginal-Isolated	2021	Yalda Rahbar Saadat	Examined how Lactococcus lactis, a vaginal probiotic, influences gene

Lactococcus lactis on the Expression of miR-21, miR-200b, and TLR-4 in CAOV-4 Cells and In Silico Revalidation			expression related to ovarian cancer progression.
Vaginal and rectal microbiome contribute to genital inflammation in chronic pelvic pain	2021	Nicole Jimenez	Studied the contribution of vaginal and rectal microbiomes to genital inflammation, with implications for gynecologic cancer risk.
The effect of the peritoneal tumor microenvironment on invasion of peritoneal metastases of high-grade serous ovarian cancer and the impact of neoadjuvant chemotherapy	2021	J. O. A.M. van Baal	Investigated how the tumor microenvironment influences peritoneal metastases in high-grade serous ovarian cancer and the role of neoadjuvant chemotherapy.

Supplementary Table S6.

A summary of the microbiota's concepts

CST Type	Dominant Microbiota	Key Features	Reported Links to Cancer Risk
**CST I**	*Lactobacillus crispatus*	Stable, low pH, protective barrier, strong lactic acid production	Considered protective; reduced dysbiosis-associated inflammation
**CST II**	*Lactobacillus gasseri*	Moderate stability, lactic acid producer but less dominant than CST I	Limited evidence; may provide moderate protection
**CST III**	*Lactobacillus iners*	Transitional state, adaptable; can coexist with dysbiosis	Potentially less protective, associated with higher susceptibility to infections
**CST IV-A (aerobic dysbiosis)**	*Gardnerella vaginalis*, *Atopobium vaginae*, facultative aerobes	Loss of Lactobacillus, aerobic bacterial overgrowth, inflammation	Linked to bacterial vaginosis, chronic inflammation, potential carcinogenesis
**CST IV-B (anaerobic dysbiosis)**	*Prevotella bivia*, *Fusobacterium nucleatum*, anaerobes	Polymicrobial community, high diversity, mucin degradation, SCFA imbalance	Associated with epithelial barrier dysfunction, immune modulation, and tumor-promoting inflammation
**CST V**	*Lactobacillus jensenii*	Acidic, protective, similar to CST I but less common	Likely protective; limited direct evidence in cancer risk
